# Using NHANES oral health examination protocols as part of an esophageal cancer screening study conducted in a high-risk region of China

**DOI:** 10.1186/1472-6831-7-10

**Published:** 2007-07-17

**Authors:** Bruce A Dye, Ru Wang, Ruth Lashley, Wenqiang Wei, Christian C Abnet, Guoqing Wang, Sanford M Dawsey, Wei Cong, Mark J Roth, Xiaojie Li, Youlin Qiao

**Affiliations:** 1 Centers for Disease Control and Prevention/National Center for Health Statistics, Hyattsville, MD, USA; 2 Dalian Medical University, School of Dentistry, Dalian, China; 3 U.S. Public Health Service, Alderson, WV, USA; 4 Cancer Institute Chinese Academy of Medical Sciences, Beijing, China; 5 National Institutes of Health/National Cancer Institute, Rockville, MD, USA

## Abstract

**Background:**

The oral health status of rural residents in the People's Republic of China has not been extensively studied and the relationship between poor oral health and esophageal cancer (EC) is unclear. We aim to report the oral health status of adults participating in an EC screening study conducted in a rural high-risk EC area of China and to explore the relationship between oral health and esophageal dysplasia.

**Methods:**

National Health and Nutrition Examination Survey (NHANES) oral health examination procedures and the Modified Gingival Index (MGI) were used in a clinical study designed to examine risk factors for esophageal cancer and to test a new esophageal cytology sampling device. This study was conducted in three rural villages in China with high rates of EC in 2002 and was a collaborative effort involving investigators from the National Institutes of Health and the Cancer Institute of the Chinese Academy of Medical Sciences.

**Results:**

Nearly 17% of the study participants aged 40–67 years old were edentulous. Overall, the mean number of adjusted missing teeth (including third molars and retained dental roots) was 13.8 and 35% had 7 contacts or less. Women were more likely to experience greater tooth loss than men. The average age at the time of first tooth loss for those with no posterior functional contacts was approximately 41 years for men and 36 years for women. The mean DMFT (decayed, missing, and filled teeth) score for the study population was 8.5. Older persons, females, and individuals having lower educational attainment had higher DMFT scores. The prevalence of periodontal disease (defined as at least one site with 3 mm of attachment loss and 4 mm of pocket depth) was 44.7%, and 36.7% of the study participants had at least one site with 6 mm or more of attachment loss. Results from a parsimonious multivariate model indicate that participants with poor oral health wemore likely to have esophageal dysplasia (OR = 1.59; 95% CI 1.06, 2.39).

**Conclusion:**

This report describes the first use of NHANES oral health protocols employed in a clinical study conducted outside of the United States. The extent and severity of poor oral health in this Chinese study group may be an important health problem and contributing factor to the prevalence of EC.

## Background

The number of comprehensive, standardized oral health examinations used in clinical studies in the People's Republic of China is unknown. Published reports of standardized oral health exams in China have been infrequent and many have been limited to surveys. Generally, research exploring associations between oral health and cancer is rare in China, with one notable exception being the assessment of oral health status of patients following radiation therapy for nasopharyngeal carcinoma [1].

Many surveys and clinical studies in China have used World Health Organization (WHO) oral health examination methods to assess for dental caries and periodontal disease [2]. In China, most adult oral health information has been collected at the regional or local level and has mainly involved urban residents from areas around Beijing, Shanghai, Chengdu, and Wuhan [3]. Lin and Schwarz reviewed literature published in English or Chinese and found that most studies were limited to assessments of the prevalence of dental caries and periodontal disease, and that some were limited by inadequate diagnostic criteria. Moreover, most studies did not include farmers, although the majority of the Chinese population is engaged in agricultural work.

The oral health status of rural residents in China has not been extensively researched. The second national oral health survey included rural adults from each of the 11 provinces chosen for survey data collection using criteria described by the WHO [4]. Rural adults were also included in a comprehensive oral health survey conducted in Guangdong Province (south China) by the University of Hong Kong in 1997 using WHO criteria and trained dental examiners [5]. Results from both studies indicated that caries experience and severe periodontal attachment loss were slightly more prevalent for adults in rural areas compared to those living in urban areas. However, an earlier survey conducted in 1990 in Guangdong Province using WHO criteria reported that gingival bleeding and the prevalence of pocket depth of 4 mm or more was greater among urban residents compared to those living in rural areas [6]. Descriptive findings from a survey conducted in the mountainous area of Dexing City, Jiangxi Province using WHO criteria indicated that caries experience and periodontal disease were more prevalent in rural areas but this disparity greatly diminished with older age [7].

Globally, "reliable epidemiological data on the periodontal health of older age groups are scarce" [8]. Although the WHO's Global Oral Data Bank is a valuable resource for geopolitical comparisons of periodontal health, it has limitations. Most periodontal information has been collected by Community Periodontal Index of Treatment Needs (CPITN) methods (now referred to as Community Periodontal Index (CPI)) using the WHO age frames of 35–44 and 65–74 years-of-age. By promoting data collection limited to these 2 decades of life using CPI, information on the adult periodontal status of other age cohorts or on attachment loss in many regions of the world is limited or unknown. Moreover, the CPI method is rarely used in the United States, reducing opportunities for more direct comparisons of periodontal status between populations in the U.S. and other countries.

The global epidemiology of dental caries among children and young adults is well known. Caries experience has declined in developed countries, and the prevalence of caries is increasing in developing countries as youth dietary patterns change [9]. However, findings for caries prevalence among adults and the elderly in the developing world have been less well reported. Although caries has historically been seen as a chronic disease of youth, older adults can also develop carious lesions [10-12]. In China and in other areas of the developing world, the formation of caries shows a gradual progression throughout life [13,14]. Although it has been reported that dental caries is the principal cause for tooth loss among adults in China [9], in non-Chinese populations aged 45 years or older, the main cause of tooth loss is less clear and may be related to periodontal disease in some regions and to dental caries in others [15-21].

Tooth loss reduces quality of life and may be related to poorer general health [22,23]. Reports from China have suggested that tooth loss may be associated with esophageal and gastric cancers [24,25] and oral cancer [26]. Although oral cancer and esophageal cancer share common risk factors, such as alcohol and tobacco use, it is unclear if poor oral health is a risk indicator for esophageal cancer.

The main aim of this paper is to describe the oral health status of a non-representative sample of adult participants in an esophageal cancer study conducted in rural Henan province, the People's Republic of China by the National Institutes of Health, National Cancer Institute (NIH/NCI) and the Cancer Institute of the Chinese Academy of Medical Sciences (CICAMS). An additional aim is to evaluate the relationship between oral health attributes and esophageal dysplasia. This paper represents the first attempt to present oral health information from data collected using the National Health and Nutrition Examination Survey (NHANES) oral health protocols in a study administered outside of the United States.

## Methods

### General study background

The NCI and the CICAMS have collaborated on studies of Esophageal Squamous Cell Carcinoma (ESCC) for over 20 years. These studies have included large intervention trials [27], screening studies [28], and other evaluations. A large number of etiologic studies have also been conducted using data from the Nutrition Intervention Trials General Population Trial cohorts, which included over 29,000 participants. One study examined the association between tooth loss at trial baseline and risk of esophageal and gastric cancer over the first 5.25 years of follow-up [24]. With over 1,000 incident cancers, the authors found significantly increased risk of upper GI cancer for persons with greater than the median number of teeth lost (n = 6), with relative risks ranging from 1.3 to 1.8 depending on the cancer site.

These collaborative studies have been conducted in Linxian (now known as Linzhou) and the surrounding counties, which have some of the highest rates of ESCC and gastric cardia cancer in the world, with age standardized incidence rates > 100/100,000 and a cumulative mortality rate of approximately 20% [29]. The current oral health exam was nested within the Cytology Sampling Study 2 (CSS2), which was designed to test the effectiveness of a novel esophageal cytology sampling device and to test methods of identifying early esophageal cancer. Subjects underwent a number of procedures and exams including esophageal cell cytology sampling, physical exam, questionnaire, oral nitrosation phenotyping, a comprehensive dental exam, and endoscopy with mucosal iodine staining and biopsy. This final procedure produces a gold-standard diagnosis to which other observations can be compared [30].

The CSS2 study was conducted in Linzhou, which is located in the Taihang mountain area of northern Henan province, during the early spring of 2002. Henan province is the most populated of the 34 administrative areas in China with a population of 92.5 million people in 2001 [31]. Nearly 977,500 people reside in the Linzhou area and 86.7% of the population is considered to be rural. Over 80% of the working population is engaged in agricultural activities and less than 8% is classified as factory or manual laborers. In Linzhou, there are 35 dentists and 4 mid-level dental providers. The nearest major urban center, An'yang, is 65 kilometers away and has a dental hospital and a health science school which provides some basic dental training for health workers.

CSS2 study participants were volunteers recruited from 3 rural Linzhou villages by the CICAMS medical team in collaboration with village doctors and local public health officials. All age-eligible subjects (adults aged 40–69 years) were invited to participate and 41%, 14%, and 25% of eligible adults were enrolled from the villages of Fentou, Jingwan, and Xifeng respectively. Study participation required participants to attend a brief morning visit at their village health center for cytology examination and a half day to visit the field station for the other exams. On the first day, participants were assessed for age eligibility, screened for contraindications to esophageal cytology and endoscopy, signed informed consent documents and were registered with a unique study identification number at their village health clinic. This was immediately followed by balloon cytology. Approximately 5 days later, participants were brought to the Beijing Medical Team field station in Yaocun Commune for a standardized health history and risk behavior interview, physical examination, biological specimen collection, endoscopy, and a standardized oral health examination. All assessments on the second examination day were performed in random order.

### General oral health procedures

Four dental examiners, 2 Chinese dentists and 2 US Public Health Service dentists, completed all of the oral health examinations. The reference examiner for this study (BD), who is also the trainer and reference examiner for the current NHANES, trained and calibrated the other three dental examiners. The dentists performed exams in alternating teams composed of 1 Chinese dentist and 1 US Public Health Service dentist with each team member rotating between the role of examiner and recorder. A trained interviewer also participated as a recorder as needed. Examiner training and calibration occurred just prior to the study and inter-examiner reliability was randomly assessed during the study by repeat dental examinations (N = 68).

Measures of oral health were performed using the same dental hand instruments (#23 Explorer, #2 Reflecting Mirror, a NIDCR Periodontal Probe) utilized for the current NHANES dental examination. The methods used were designed to be compatible with existing NHANES criteria including performing the periodontal assessment on 2 randomly selected quadrants (one maxillary and one mandibular), which has been described in detail elsewhere [32-35]. All instruments were sterilized with a portable steam autoclave. All examinations were performed indoors using artificial light. Participates were examined in portable dental chairs in a recumbent position.

Two questionnaires, a medical questionnaire and a dental questionnaire, were administered by 3 trained interviewers who had at least a secondary education and who spoke the local dialect. The purpose of the medical questionnaire was to identify study participants who should be excluded from portions of the oral health examination for their personal safety. Individuals receiving a medical exclusion were exempted from the root caries and loss of attachment assessments. The dental questionnaire consisted of 2 questions related to dental pain and 2 questions concerning tooth loss that were used on the baseline questionnaire of the Nutrition Intervention Trial.

### Study population

Subjects in this study were adult volunteers aged 40–69 years-old from 3 villages surrounding Linzhou, People's Republic of China who participated in the CSS2 study. This study, including the oral health exam, was approved by the Institutional Review Boards of the NCI and the CICAMS. All study participants gave informed consent. Oral health examination data was obtained from 740 participants aged 40–67 years-old for this report. We excluded 22 persons with missing histology information to produce an analytical sample of 718 dentate and edentulous adults. For a separate analytical sample of only dentate adults, we excluded 125 individuals who were identified as edentulous during the examination, 18 individuals who had incomplete periodontal examination record, and 18 persons with missing histology information, to yield 579 dentate adults. Reasons for not having a complete periodontal record included exclusion for medical reasons or having a remaining dentition that was not assessable, such as having only retained dental root fragments.

### Dentition evaluation

The tooth count assessment involved examining the maxillary and mandibular arches to identify the presence or absence of permanent teeth. All teeth, including third molars, were assessed. Missing teeth were identified as not present regardless of reason. Permanent retained dental roots were identified separately. Dentate status inter-rater reliability was considered to be excellent with Kappa statistics > 0.90 and percent agreements > 94%. For the occluding pairs assessments, the Kappa statistics ranged from 0.79–0.85 with percent agreements > 93%.

A person was designated "edentulous" if all 32 tooth spaces were not occupied by at least one natural tooth or retained dental root. We defined "functional edentulism" has having any combination of missing and retained dental roots and this was determined post data collection. The "adjusted missing" teeth were calculated by the sum of the observed missing teeth and the number of residual dental roots present for all 32 tooth spaces. Removable prosthetic status was derived from data collected in the coronal caries assessment. A replacement was considered to exist if it was visible in the mouth or if a study participant reported that one existed regardless of frequency of use.

Functional occlusal contacts were assessed using methods derived from an examination utilized in the United Kingdom [36] and implemented on the 2003–2004 NHANES. The occlusal contacts examination consisted of an assessment of the posterior occluding tooth pairs and a total count of the number of anterior contacts present. The examiner recorded the distribution of posterior contacts by assessing for the presence of a contact if the contact formed a vertical occlusal stop in the eligible "zone." There were 8 "zones" in each of the posterior segments (right and left) beginning distal to the cuspid. Each of the premolars was assessed as a single zone and the molars were counted as two zones each. All natural and prosthetic teeth were eligible for assessment. For this analysis, the number of occluding tooth pairs was derived. To approximate the 1^st^, 2^nd^, and 3^rd ^molar areas, a pair was considered present if a contact was present in either zone 3 or 4, in zones 5 or 6, and in zones 7 or 8 respectively. Zones 1 and 2 were counted individually. The algorithm produced a count of up to 5 occluding pairs for each side (i.e., the right and left sides).

### Dental caries examination

The NHANES diagnostic criteria for dental caries are historically referred to as the "Radike" method and were based upon guidelines developed from the Proceedings of the Conference on the Clinical Testing of Cariostatic Agents in 1968 [37]. All teeth except the third molars were examined with a visual-tactile method using a #23 dental explorer. All participants were required to rinse with water prior to the oral health examination and remaining food debris covering tooth surfaces were removed with 2 × 2 sponges and the explorer. Examinations were performed with artificial lighting with the study participants in a supine position. The Inter-rater reliability for caries assessment was considered to be very good with Kappa statistics ranging from 0.85–0.94 and percent-agreements ranging from 88%–97% for the various caries measures assessed.

Residual root surfaces were counted but were not assessed for caries. Retained roots were classified as a permanent residual tooth structure if more than 90% of the coronal structure had been destroyed by caries. For purposes of computing DMFT (decayed, missing, and filled teeth) scores [38], a retained root was considered to have either 4 carious surfaces if it was an anterior tooth or 5 carious surfaces if it was a posterior tooth. Assessments of root caries and restorations indicated whether one or more lesions (restorations) were present in the mouth and were recorded as a "whole-mouth" dichotomous score for each condition. The explorer was used to confirm softness in the root surface lesion. Root lesions with hardened or stained root structure were not assessed as carious.

Missing teeth were subclassified according to two general causes of tooth loss: (1) extraction due to caries or periodontal disease or (2) extraction due to other reasons such as trauma. Moreover, the status of replacement for missing teeth was assessed and included three options: missing but not replaced, replaced with a removable restoration, or replaced with a fixed restoration. A replacement was considered to exist if it was visible in the mouth or if the study participant reported that one existed, regardless of frequency of use. Teeth coded as missing for reasons other than disease were not included in the computation of DMFT scores.

### Periodontal examination

The Modified Gingival Index (MGI) was used to visually assess for gingival inflammation independent of dental probing. Four sites for each eligible tooth were evaluated following diagnostic criteria described by Lobene and coworkers [39]. Scoring began with the distal-facial site of the 2^nd ^molar in the randomly selected maxillary quadrant. The evaluation proceeded to the mid-facial site and then to the mesial-facial site of the 2^nd ^molar with the lingual site assessed last. The examination proceeded from posterior to anterior until the central incisor was assessed.

The periodontal examination followed the MGI assessment and included measurements to determine the loss of clinical attachment and the identification of bleeding from probing. The distal, mid-facial, and mesial periodontal sites were assessed with a color-coded periodontal probe (NIDCR probe) graduated at 2, 3, 6, 8, 10, and 12 millimeters. The presence or absence of bleeding following probing was noted by the examiner for each site probed. Inter-rater reliability statistics (Kappa) ranged from 0.49–0.83 for assessing percent of sites with loss of attachment => 3 mm and ranged from 0.33–0.67 for assessing periodontal disease prevalence. The inter-examiner percent-agreements for these periodontal conditions ranged from 81%–95% and from 80%–87% respectively.

Periodontal disease was defined at a lower threshold as 1 or more sites with attachment loss of 3 mm or greater and a pocket depth of 4 mm or greater, and this was based on previously reported research [40]. We also defined a higher threshold of periodontal disease as 1 or more sites with attachment loss of 4 mm or greater and a pocket depth of 5 mm or greater. The reasoning for including this higher threshold is explained in the "Discussion" section. A subject-level MGI score was determined by selecting the highest 2-same-scores following previously described guidelines [39].

### Data analysis

Demographic and smoking data were obtained from the main health history questionnaire. These included information on age, gender, place of residence, education attainment, and family history of cancer. Participants were categorized as having any or no histologically proven esophageal squamous dysplasia. Additional details describing esophageal biopsy techniques and histology categories are described elsewhere [25].

STATA software (Version 9.2 SE; StataCorp, College Station, TX) was used to perform all statistical analyses. Analysis of variance (ANOVA) was used to test mean scores and proportions were compared using Chi-square tests. Differences were considered to be statistically significant when the *p *value was less than 0.05. To assess the relationship between the presence of dysplasia and the covariates using logistic regression models, a "poor oral health" variable was calculated. Poor oral health was defined as having a mean attachment loss (AL) greater than the median (>2.4 mm) and having a mean DMFT score greater than the median (>9). Parsimonious models were determined by covariate exclusion with criteria for inclusion set at *p *< 0.05. We assessed for potential interactions throughout the modeling process but no significant interactions were found.

## Results

Participants who were older, female, less educated, and residents of Jingwan were more likely to have missing teeth or to be edentulous (Table 1). The prevalence of edentulism was 17% in this study group. Nearly 18% were functionally edentulous. More than 29% of residents from Jingwan were functionally edentulous compared to 12.7% of people residing in Xifeng. Individuals aged 56–67 years-of-age were more than twice as likely to be functionally edentulous compared to those 40–56 years of age. The mean number of retained dental roots was 1.3 per participant and the adjusted mean number of missing teeth was 13.8 when third molars were assessed.

**Table 1:** The percent (%) prevalence of edentulism and the mean and standard errors (SE) for tooth loss by selected characteristics for study participants: Linzhou, People's Republic of China, 2002.

**Characteristic**	**Edentulous** **(%) (SE)**	**Functional Edentulous^**$ **^** **(%) (SE)**	**28 Teeth Missing** **mean (SE)**	**32 Teeth Missing** **mean (SE)**	**32 Teeth Residual Roots** **mean (SE)**	**32 Teeth Adjusted Missing^**# **^** **mean (SE)**
Age						
56–67 years old	24.4 (2.5)*	26.4 (2.5)*	13.2 (0.6)*	16.2 (0.7)*	1.3 (0.1)	17.5 (0.6)*
40–55 years old^R^	11.7 (1.5)	11.9 (1.6)	7.6 (0.4)	10.0 (0.5)	1.3 (0.1)	11.3 (0.5)
Gender						
Women	19.9 (1.9)*	21.3 (1.9)*	10.9 (0.5)*	13.7 (0.6)*	1.4 (0.1)	15.1 (0.5)*
Men^R^	12.8 (1.9)	13.1 (1.9)	8.6 (0.5)	11.0 (0.6)	1.1 (0.1)	12.1 (0.5)
Village						
Jingwan	27.0 (3.4)*	29.4 (3.5)*	13.6 (0.9)*	16.5 (0.6)*	1.3 (0.2)	17.8 (0.9)*
Fentou	15.7 (2.3)	16.5 (2.4)	8.9 (0.7)	11.4 (0.7)	1.3 (0.2)	12.8 (0.7)
Xifeng^R^	12.4 (1.8)	12.7 (1.8)	8.9 (0.6)	11.4 (0.9)	1.3 (0.1)	12.7 (0.6)
Education						
Did not complete Primary	20.4 (2.2)*	22.0 (2.3)*	11.7 (0.6)*	14.5 (0.6)*	1.4 (0.1)	15.9 (0.6)*
Completed Primary School^R^	14.1 (1.7)	14.6 (1.7)	8.5 (0.5)	11.0 (0.5)	1.2 (0.1)	12.2 (0.5)
Smoking						
Has smoked cigarettes	16.2 (2.6)	16.8 (2.7)	9.7 (0.7)	12.1 (0.8)	1.0 (0.1)*	13.1 (0.8)
Never smoked cigarettes^R^	17.1 (1.6)	18.2 (1.7)	10.0 (0.4)	12.7 (0.5)	1.4 (0.1)	14.1 (0.5)
Family history of any cancer						
Yes	16.4 (2.0)	17.6 (2.1)	9.9 (0.6)	12.5 (0.6)	1.0 (0.1)*	13.5 (0.6)
No^R^	17.3 (1.9)	18.0 (1.9)	10.0 (0.5)	12.6 (0.6)	1.5 (0.1	14.1 (0.5)
Had any dysplasia						
Yes	15.2 (2.3)	17.3 (2.5)	10.6 (0.7)	13.3 (0.7)	1.1 (0.1)	14.4 (0.7)
No^R^	17.7 (1.7)	17.9 (1.7)	9.5 (0.5)	12.1 (0.5)	1.4 (0.1)	13.5 (0.5)
Total	17.1 (1.4)	17.8 (1.4)	9.9 (0.4)	12.5 (0.4)	1.3 (0.1)	13.8 (0.4)

Data Source: CSS2

N = 718

Notes:

(R): Reference category

(*): *p *< 0.05 compared to the Reference Category.

(#): Adjusted Missing is the sum of the observed number of missing teeth (32) and number of residual roots (32) to yield an estimate of Total "Functional" Missing Teeth.

($): Functional Edentulous defined as having either fully missing teeth and/or residual roots present.

**Figure 1:**
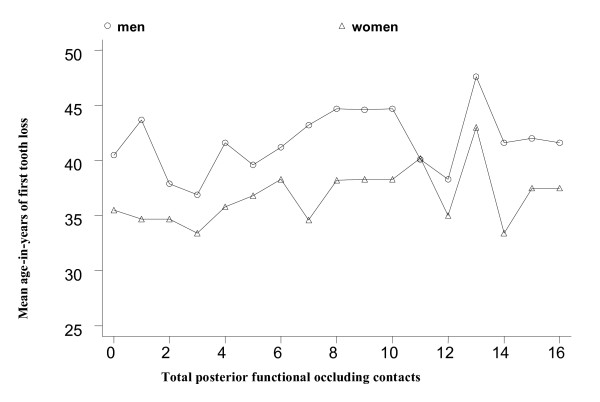
The mean age-in-years of first tooth loss of study participants with posterior functional contacts by gender: Linzhou, People's Republic of China, 2002.

This Linzhou study group had approximately 18 teeth per person when all 32 teeth were accounted for (Table 2). When excluding third molars, the mean number of teeth was nearly 17 per person. Approximately 11% had all of their natural teeth excluding third molars. Individuals aged 56 years or older averaged 6 fewer teeth than those who were younger and were less likely to have all of their natural teeth. Residents of Fentou were more likely to have all of their natural teeth than those living in Xifeng (15.7% vs. 9.3%) and only 5.9% of persons living in Jingwan had all of their natural teeth. Compared to men, women were less likely to have retained all of their natural teeth (13.7% vs. 8.5%). Approximately 40% of the study participants had 7 or fewer functional tooth contacts. Persons aged 56 years or older, not completing primary school, women, and those not living in Fentou were more likely to have fewer posterior contacts. Among all study participants, the average age at the time of first tooth loss was 39 years. The average age at the time of first tooth loss for those with no posterior functional contacts was approximately 41 years for men and 36 years for women (Figure 1).

**Table 2:** The percent (%) prevalence, mean and standard errors (SE) for tooth retention, posterior dental contacts and age of first tooth loss by selected characteristics for study participants: Linzhou, People's Republic of China, 2002.

**Characteristic**	**32 Teeth** **mean (SE)**	**28 Teeth** **mean (SE)**	**28 Teeth Present** **(%) (SE)**	**Posterior Contacts** **mean (SE)**	**7 or less Contacts** **(%) (SE)**	**Age first Tooth loss^**+ **^** **mean (SE)**
Age						
56–67 years old	14.5 (0.7)*	13.7 (0.6)*	5.6 (1.3)*	8.3 (0.3)	36.8 (2.8)	40.8 (0.7)*
40–55 years old^R^	20.7 (0.5)	19.4 (0.4)	14.2 (1.7)	8.9 (0.2)	32.8 (2.2)	38.3 (0.5)
Gender						
Women	16.9 (0.5)*	16.0 (0.5)*	8.5 (1.3)*	8.4 (0.2)*	33.1 (2.7)	37.1 (0.5)*
Men^R^	19.9 (0.6)	18.5 (0.5)	13.7 (1.9)	9.1 (0.3)	35.4 (2.3)	41.9 (0.6)
Village						
Jingwan	14.5 (0.9)*	13.7 (0.8)*	5.9 (1.8)	8.4 (0.3)*	35.3 (3.7)	38.4 (0.8)*
Xifeng	19.3 (0.7)	18.0 (0.5)	15.7 (2.3)*	8.2 (0.2)*	37.9 (2.7)*	38.6 (0.6)*
Fentou ^R^	19.3 (0.6)	18.1 (0.5)	9.3 (1.6)	9.4 (0.3)	29.4 (2.9)	40.6 (0.7)
Education						
Did not complete Primary	16.1 (0.6)*	15.2 (0.6)*	7.1 (1.4)*	8.1 (0.3)*	37.2 (2.7)	37.2 (0.6)*
Completed Primary School^R^	19.8 (0.5)	18.6 (0.5)	13.4 (1.7)	9.1 (0.2)	32.4 (2.3)	40.7 (0.5)
Smoking						
Has smoked cigarettes	18.9 (0.8)	17.5 (0.7)	12.7 (2.3)	9.2 (0.4)	32.1 (3.3)	41.6 (0.7)*
Never smoked cigarettes^R^	17.9 (0.5)	16.9 (0.4)	9.9 (1.2)	8.5 (0.2)	35.4 (2.1)	38.4 (0.5)
Family history of any cancer						
Yes	18.5 (0.6)	17.3 (0.6)	10.9 (1.7)	9.0 (0.2)*	31.5 (2.6)	39.0 (0.6)
No^R^	17.9 (0.5)	16.9 (0.5)	10.5 (1.5)	8.4 (0.2)	36.8 (2.4)	39.4 (0.5)
Had any dysplasia						
Yes	17.6 (0.7)	16.6 (0.7)	9.1 (1.8)	8.6 (0.2)	34.5 (2.2)	39.3 (0.5)
No^R^	18.5 (0.5)	17.4 (0.5)	11.1 (1.4)	8.6 (0.3)	35.6 (3.1)	39.2 (0.7)
Total	18.2 (0.4)	17.1 (0.4)	10.7 (1.1)	8.6 (0.2)	34.9 (1.8)	39.2 (0.4)

Data Source: CSS2

N = 718

Notes:

(R): Reference category

(*): *p *< 0.05 compared to the Reference Category.

(+):Age in years of first permanent tooth loss

The mean DMFT and DFT (decayed and filled teeth) scores are presented in Table 3. The mean DMFT for the study population was 8.0. Persons 56 years-of-age or older were more likely to have a higher DMFT compared to younger study participants (9.8 vs. 7.0) and participants from Jingwan village were more likely to have higher DMFT scores compared to those from Fentou (9.4 vs.7.1). Females, having lower educational attainment, and having esophageal dysplasia were more likely to have a higher DMFT score as well. The mean DFT score for the study population was 2.5 and women were more likely to have a higher DFT compared to men (2.8 vs. 2.1). For both men and women, the majority of carious teeth were untreated or had recurrent decay (92.7% and 94.1% respectively). Slightly less than half of the participants had root caries (47.9%). Participants from Xifeng village were more likely to have root caries compared to participants from Fentou (54.9% vs. 42.9%).

**Table 3:** The percent (%) prevalence, mean and standard errors (SE) for dental caries experience by demographic, dysplasia, and tooth loss status for study participants: Linzhou, People's Republic of China, 2002.

**Characteristic**	**Sound Teeth** **mean (SE)**	**DMFT Score** **mean (SE)**	**DFT Score** **mean (SE)**	**DMFS Score** **mean (SE)**	**DFS Score** **mean (SE)**	**DS/DFS** **(%) (SE)**	**Root Caries** **(%) (SE)**
Age							
56–67 years old	17.2 (0.5)*	9.8 (0.5)*	2.9 (0.3)*	42.8 (2.3)*	10.3 (1.0)*	94.9 (1.5)	49.2 (3.4)
40–55 years old^R^	20.3 (0.3)	7.0 (0.4)	2.3 (0.1)	30.2 (1.4)	7.8 (0.5)	92.7 (1.4)	47.1 (2.6)
Gender							
Women	18.6 (0.4)*	8.6 (0.4)*	2.8 (0.2)*	37.0 (1.7)*	9.7 (0.7)*	94.1 (1.2)	50.5 (2.7)
Men^R^	19.9 (0.4)	7.2 (0.4)	2.1 (0.2)	32.0 (1.8)	7.5 (0.7)	92.7 (1.8)	44.8 (3.0)
Village							
Jingwan	17.6 (0.7)*	9.4 (0.7)*	2.5 (0.3)	41.7 (3.0)*	8.9 (1.1)	91.8 (2.7)	40.3 (4.5)
Xifeng	19.1 (0.4)	8.0 (0.4)	2.5 (0.2)	34.5 (1.8)	8.5 (0.7)	96.0 (1.1)*	54.9 (3.0)*
Fentou ^R^	20.2 (0.5)	7.1 (0.4)	2.5 (0.2)	30.9 (2.0)	8.7 (0.9)	91.1 (2.2)	42.9 (3.4)
Education							
Did not complete Primary	18.0 (0.5)*	9.1 (0.5)*	2.8 (0.2)*	39.8 (2.1)*	10.0 (0.8)*	93.5 (1.6)	54.5 (3.2)*
Completed Primary School^R^	20.0 (0.4)	7.2 (0.4)	2.3 (0.2)	31.3 (1.5)	7.9 (0.6)	93.7 (1.6)	43.4 (2.6)
Smoking							
Has smoked cigarettes	19.5 (0.6)	7.4 (0.5)	2.0 (0.2)*	32.6 (2.4)	7.0 (0.8)*	91.0 (2.5)	44.7 (3.9)
Never smoked cigarettes^R^	19.0 (0.3)	8.2 (0.3)	2.7 (0.2)	35.5 (1.5)	9.3 (0.6)	94.4 (1.1)	49.1 (2.4)
Family history of any cancer							
Yes	19.1 (0.4)	8.0 (0.4)	2.3 (0.2)	34.6 (1.9)	7.6 (0.6)*	92.4 (1.6)	41.2 (3.0)*
No^R^	19.2 (0.4)	8.0 (0.4)	2.7 (0.2)	34.8 (1.7)	9.6 (0.7)	94.5 (1.3)	53.4 (2.8)
Had any dysplasia							
Yes	18.5 (0.5)	8.7 (0.5)*	2.3 (0.2)	37.8 (2.3)	9.2 (0.7)	93.2 (1.9)	50.0 (3.6)
No^R^	19.5 (0.4)	7.7 (0.3)	2.6 (0.2)	33.3 (1.5)	7.9 (0.7)	93.8 (1.2)	47.0 (2.5)
Missing teeth category							
(Q1) 12–31 teeth	9.1 (0.4)*	17.4 (0.5)*	1.9 (0.2)	78.5 (2.2)*	6.2 (0.8)	89.8 (3.1)	44.3 (3.8)
(Q2) 7–11 teeth	17.4 (0.3)*	9.5 (0.3)*	3.6 (0.3)*	41.2 (1.4)*	12.8 (1.1)*	93.6 (1.8)	56.7 (3.8)*
(Q3) 4–6 teeth	22.5 (0.3)*	5.0 (0.3)*	2.6 (0.2)*	20.4 (1.2)*	9.1 (0.9)*	95.6 (1.7)	48.3 (4.1)
(Q4) 0–3 teeth^R^	25.3 (0.2)	2.4 (0.2)	1.8 (0.2)	9.0 (0.9)	6.2 (0.8)	94.1 (2.1)	44.3 (3.8)
Total	19.2 (0.3)	8.0 (0.3)	2.5 (0.1)	34.7 (1.2)	8.7 (0.5)	93.6 (1.0)	47.9 (2.0)

Data Source: CSS2

N = 579

Notes:

(R): Reference category

(*): *p *< 0.05 compared to the Reference Category.

The CSS2 study group had a mean attachment loss of 2.8 mm (Table 4). Mean attachment loss was more likely to be greater among men, cigarette smokers, and participants residing in Jingwan and Xifeng villages. Mean attachment loss appeared to increase incrementally with each higher category of tooth loss. Men, smokers, and persons residing in Jingwan and Xifeng had more sites of attachment loss at 4 mm+ compared to women, non-smokers, and residents of Fentou. The mean MGI score was 2.75. Younger study participants and residents from Jingwan and Xifeng were more likely to have higher mean MGI scores compared to those 56 years-of-age and older or living in Fentou village. Nearly 45% of the study participants had periodontal disease at a lower threshold, whereas 17.8% had disease at an upper diagnostic threshold. Periodontal disease at the upper threshold was significantly more prevalent among residents from Xifeng compared to individuals living in Fentou (21.5% vs. 13.3%).

**Table 4:** The percent (%) prevalence, mean and standard errors (SE) for periodontal status indicators by demographic, dysplasia, and tooth loss status for study participants: Linzhou, People's Republic of China, 2002.

**Characteristic**	**Attachment Loss (mm)** **mean (SE)**	**# sites AL≥4 mm** **mean (SE)**	**AL>=6 mm** **% (SE)**	**Periodontitis Lower^**1 **^** **% (SE)**	**Periodontitis Higher^**2 **^** **% (SE)**	**MGI Score** **mean (SE)**
Age						
56–67 years old	2.89 (0.11)	6.9 (0.5)	39.4 (3.4)	40.8 (3.3)	14.6 (2.4)	2.64 (0.05)*
40–55 years old^R^	2.70 (0.07)	7.0 (0.4)	36.3 (2.5)	46.9 (2.5)	19.5 (2.0)	2.81 (0.03)
Gender						
Women	2.52 (0.07)*	5.5 (0.3)*	29.7 (2.5)*	42.2 (2.7)	17.1 (2.1)	2.71 (0.04)
Men^R^	3.07 (0.10)	8.8 (0.5)	46.8 (3.0)	47.8 (3.0)	18.5 (2.3)	2.79 (0.04)
Village						
Jingwan	3.20 (0.15)*	9.1 (0.7)*	47.9 (4.6)*	48.8 (4.6)*	16.8 (3.4)	2.59 (0.08)*
Xifeng	2.92 (0.09)*	7.5 (0.5)*	41.2 (3.0)*	46.9 (3.0)*	21.5 (2.4)*	2.73 (0.04)*
Fentou ^R^	2.31 (0.08)	5.0 (0.4)	26.1 (3.1)	39.4 (0.3)	13.3 (2.4)	2.86 (0.05)
Education						
Did not complete Primary	2.82 (0.10)	6.6 (0.5)	37.2 (3.1)	44.2 (3.1)	14.5 (2.2)	2.69 (0.05)
Completed Primary School^R^	2.73 (0.08)	7.2 (0.4)	37.6 (2.6)	45.1 (2.6)	20.0 (2.1)	2.79 (0.04)
Smoking						
Has smoked cigarettes	3.15 (0.12)*	8.9 (0.6)*	46.3 (4.0)*	50.3 (3.9)	18.0 (3.0)	2.70 (0.06)
Never smoked cigarettes^R^	2.63 (0.07)	6.3 (0.3)	34.2 (2.3)	42.7 (2.3)	17.7 (1.8)	2.76 (0.03)
Had any dysplasia						
Yes	2.83 (0.12)	6.8 (0.5)	35.6 (2.4)	40.9 (3.6)	17.7 (2.8)	2.76 (0.06)
No	2.72 (0.07)	7.0 (0.4)	38.9 (3.6)	45.3 (2.5)	17.3 (1.9)	2.73 (0.04)
Family history of any cancer						
Yes	2.77 (0.09)	6.7 (0.4)	39.4 (3.0)	46.5 (3.0)	19.3 (2.4)	2.73 (0.05)
No	2.76 (0.08)	7.2 (0.4)	35.8 (2.7)	43.3 (2.7)	16.5 (2.1)	2.76 (0.04)
Number of missing teeth						
(Q1) 12–31 teeth	3.74 (0.19)*	6.4 (0.6)	53.7 (4.6)*	38.0 (4.4)	16.5 (3.4)	2.32 (0.10)*
(Q2) 7–11 teeth	2.97 (0.10)*	8.9 (0.6)*	46.0 (3.9)*	52.4 (3.9)	24.4 (3.3)*	2.85 (0.05)
(Q3) 4–6 teeth	2.42 (0.09)*	7.1 (0.7)*	29.7 (3.8)	41.4 (4.1)	13.8 (2.9)	2.84 (0.05)
(Q4) 0–3 teeth^R^	2.16 (0.07)	5.3 (0.5)	24.0 (3.3)	44.9 (3.8)	15.6 (2.8)	2.87 (0.05)
Total	2.77 (0.06)	7.0 (0.3)	36.7 (2.0)	44.7 (2.0)	17.8 (2.0)	2.75 (0.03)

Data Source: CSS2

N = 579

Notes:

(1): Periodontal Disease defined as at least one site with 3 mm Attachment Loss and 4 mm Pocket Depth

(2): Periodontal Disease defined as at least one site with 4 mm Attachment Loss and 5 mm Pocket Depth

(R): Reference category

(*): *p *< 0.05 compared to the Reference Category

Table 5 shows the prevalence of dysplasia by selected demographic and smoking characteristics and the results from logistic regression modeling. Approximately 32% of the study group had esophageal squamous dysplasia. Among individuals with poor oral health or 12–31 missing teeth, 40.2% and 38.5% respectively had dysplasia. Unadjusted model results indicate that esophageal dysplasia was significantly associated with a family history of cancer, missing 12–31 teeth and having poor oral health. In a model that included all of the covariates, only a family history of cancer was significantly associated with dysplasia. However, when non-significant covariates were removed to produce the most parsimonious model, both a family history of cancer (OR = 1.55; 95% CI 1.09, 2.21) and poor oral health (OR = 1.59; 95% CI 1.06, 2.39) were significantly associated with dysplasia.

**Table 5:** The number, percent (%), standard error (SE), odds ratios (OR) and 95% confidence intervals (CI) for the presence of dysplasia among dentate study participants aged 40–69 years: Linzhou, People's Republic of China, 2002

**Characteristic**	**Dysplasia**		**Unadjusted**		**Adjusted Model**^**2**^		**Adjusted Model**^**3**^	
	**N**	**% (SE)**	**OR**	**(CI)**	**OR**	**(CI)**	**OR**	**(CI)**
Age								
56–67 years old	208	34.6 (3.3)	1.19	(0.83, 1.71)	1.10	(0.75, 2.76)		-
40–55 years old^R^	371	30.7 (2.4)	1.00		1.00			
Gender								
Women	319	30.1 (2.6)	1.22	(0.87, 1.74)	1.08	(0.65, 1.77)		-
Men^R^	260	34.6 (3.0)	1.00		1.00			
Village								
Jingwan	114	38.6 (4.6)	1.24	(0.77, 2.00)	1.11	(0.68, 1.82)		
Xifeng	266	28.2 (2.8)	0.77	(0.52, 1.15)	0.73	(0.49, 1.11)		-
Fentou ^R^	199	33.7 (3.4)	1.00		1.00			
Education								
Did not complete Primary	235	28.5 (3.0)	0.75	(0.53, 1.08)	0.70	(0.53, 1.18)		-
Completed Primary School^R^	344	34.6 (2.6)	1.00		1.00			
Smoking								
Has smoked cigarettes	158	35.4 (3.8)	1.23	(0.84, 1.81)	1.12	(0.66, 1.89)		-
Never smoked cigarettes^R^	421	30.9 (2.3)	1.00		1.00			
Family history of any cancer								
Yes	263	37.3 (3.0)*	1.54	(1.08, 2.18)*	1.52	(1.06, 2.18)*	1.55	(1.09, 2.21)*
No^R^	316	27.8 (2.5)	1.00		1.00		1.00	
Number of missing teeth								
(Q1) 12–31 teeth	117	38.5 (4.5)*	1.70	(1.03, 2.83)*	1.45	(0.76, 2.76)		
(Q2) 7–11 teeth	161	33.5 (3.7)	1.38	(0.85, 2.21)	1.36	(0.81, 2.26)		-
(Q3) 4–6 teeth	137	31.4 (4.0)	1.25	(0.76, 2.06)	1.31	(0.78, 2.18)		
(Q4) 0–3 teeth^R^	164	26.8 (3.5)	1.00		1.00			
Poor oral health^1^								
Yes	132	40.2 (4.3)*	1.58	(1.06, 2.37)*	1.37	(0.82, 2.26)	1.59	(1.06, 2.39)*
No^R^	447	29.8 (2.2)	1.00		1.00		1.00	
Total	579	32.1 (1.9)		-		-		-

Data Source: CSS2

Notes:

(R): Reference category

(*): *p *< 0.05 compared to the Reference Category

(1): Poor oral health defined as having mean AL measure greater than median (>2.4 mm) and having mean DMFT score greater than median (>9)

(2): Adjusted Model is a full model, includes all covariates

(3): Adjusted Model is the most parsimonious model, includes only statistically significant covariates

## Discussion

It has been reported that major studies in China examining tooth loss have been "uncommon and mainly conducted in urban areas," and have generally involved the elderly [3]. A survey from Chengdu city reported that 19.7% of persons aged 60 years or older were edentulous and only 4.8% had all of their natural 28 teeth [41]. Although the mean number of missing teeth among rural residents aged 65–74 years was 11.6 in a south China survey, only 3.4% were edentulous [42]. Findings from the 2^nd ^Chinese National Oral Health Survey indicated that 10.5% of participants aged 65–74 years were edentulous, with rural residents retaining 16.8 natural teeth compared to 18.8 natural teeth for persons living in urban areas [4]. Based on 28 natural teeth, tooth retention for this rural Linzhou study group was very similar to the national estimates for rural residents (17.1 vs. 16.8). Although tooth retention was similar in magnitude to national estimates in China, the prevalence of edentulism in the Linzhou study group for 56–67 year-olds was greater (16.3%).

Edentulism among the Linzhou study participants was significantly lower in 40–55 year-olds compared to the 56–67 year-olds (11.7% vs. 24.4%). It has been reported that edentulism in the Beijing area was "rarely seen" in persons younger than 60 years-old, and in Hong Kong it was "not apparent until after age 55" and was seen at a much reduced rate compared to many regions of the developed world [43,44]. Complete tooth loss (approximately 17%) was higher in this Chinese study group than recent reports from the United States, which showed that only 5% of persons aged 40–59 years were edentulous [45].

Older adults who are cigarette smokers are more likely to have greater tooth loss, fewer functional contacts, and greater tooth loss due to periodontal disease [46-48]. In the United States, it is estimated that among current smokers, nearly 75% of periodontal disease is attributable to smoking [49]. Findings from a 1996 national survey in China indicate that smoking was more prevalent among men (63%) compared to women (3.8%) [50]. Approximately 20% of the Linzhou study group was identified as current smokers and among those, 98% were men (data not shown). We found that the association between smoking status and the mean number of retained dental roots was statistically significant, but not clinically relevant (1.0 retained roots for smokers vs. 1.4 retained roots for non-smokers). Smoking status was not associated with the calculated mean number of adjusted missing teeth in this study group of 40–67 year-olds. Furthermore, unlike in the United States, we found that smoking was not associated with edentulism and tooth loss in a dentition excluding third molars.

Findings from the Linzhou study group showed that women, older individuals, and persons with lower educational attainment had higher DMFT scores. The mean DMFT scores for men and women with dental caries in this study group were 7.6 and 9.3 respectively. Results for 35–44 year-olds and 65–74 year-olds who participated in the 2^nd ^National Oral Health Survey in China were 1.7 and 11.6 for men and 2.6 and 13.0 for women respectively [4]. Overall, our findings suggest that women from the Linzhou study population experience more coronal and root decay compared to men, and women are more likely to have retained residual dental roots and more teeth decayed or filled.

Survey results from the 2^nd ^National OH Survey and a comprehensive oral health survey conducted in Guangdong Province in 1997 indicated that caries experience was slightly more prevalent for adults in rural areas compared to those living in urban areas [4,5]. Overall, findings from the 2^nd ^National OH Survey indicated that the prevalence of dental caries among rural Chinese was 60% for 35–44 year-olds and 64% for 65–74 year-olds [4]. The prevalence of dental caries among this rural study group aged 40–67 years was approximately 68%.

Lin and coworkers reported that studies measuring root surface caries are quite uncommon in China [51]. They reported a prevalence of 38% among the 65–74 year-olds, whereas findings from 56–67 year-olds in our Linzhou study group showed a prevalence of 48.5%. Chinese studies reporting mean residually retained roots are even rarer. Lo and coworkers reported a mean retention of 0.5 residual dental roots for 65–74 year-olds residing in Hong Kong [52], whereas the findings in 56–67 year-olds in our study showed a mean retention of 1.4 dental roots. It should be noted that differences exist between the WHO and NHANES methodologies for classifying dental roots.

From 1975–76, a study was conducted on 1084 adults aged 21–50 years-old and 1400 school-aged children in Linxian (now known as Linzhou) to examine the relationship between dental caries and urine fluoride levels. They reported that dental caries prevalence was high (80.7%) and that oral hygiene was poor among the adults examined [53]. Because the methodologies used in this earlier Chinese study are not comparable to the WHO or the NHANES caries assessment protocols, comparisons are very problematic. The caries diagnostic methods described assessed for caries using a 5-point graduated scale with "primary caries" limited only to caries in enamel. As the diagnostic criteria increased along this scale, caries in level 4 were defined as deep dentinal caries with likely pulpal involvement and level 5 caries were limited to retained dental roots.

General observations from the Linzhou study group showed that men, older individuals, persons with higher educational attainment, and smokers had worse periodontal health. Using a CPITN/CPI score of "4" as reflective of an advanced periodontal pocket (i.e., 6 mm or greater), studies conducted in China within the past decade have reported the percentage of individuals with at least one advanced periodontal pocket to range from 4–25% among 65–74 year-olds living in Shanghai, Hong Kong, Hubei, and Guangdong [54-57]. Findings from the 2^nd ^National Oral Health Survey indicated that 3.8% of Chinese aged 65–74 years had advanced periodontal pocketing [4]. In the Linzhou study group, the prevalence of periodontal pockets measured at 6 mm or greater among persons aged 56–67 years-old was 9.8% (data not shown).

Using the WHO attachment loss categories [2], Corbet and coworkers reported that among those 65–74 years-of-age, 55% of rural persons and 48% of urban persons had at least one site of attachment loss measured at 6 mm or more [57]. Moreover, the authors reported that unlike advanced periodontal pocket findings, attachment loss among this older age group was consistent with findings from Hong Kong and Beijing. More than 39% of the CSS2 participants from rural Linzhou aged 56–67 years-old were found to have at least one site with 6 mm or more of attachment loss.

The periodontal health status of rural residents in China has not been extensively researched. Rural adults were included in a comprehensive oral health survey conducted in Guangdong Province in 1997 using WHO criteria [5]. Results indicated that among rural adults, 42% of 35–44 year-olds had considerable attachment loss for age (minimum 2 sextants w/4+ mm) and 38% of 65–74 year-olds had considerable attachment loss for age (minimum 2 sextants w/6+ mm) [54]. Moreover, attachment loss was slightly more prevalent for adults in rural areas compared to those living in urban areas. However, an earlier survey conducted in 1990 in Guangdong Province using WHO criteria reported that the prevalence of pocket depth of 4 mm or more was greater among urban residents compared to those living in rural areas [7]. Results from the 2^nd ^National Oral Health Survey showed that 14% of rural 35–44 year-olds had "shallow" pocket depths (4–5 mm) and 2.2% had "deep" pockets (6+ mm)[4]. Moreover, the pocket depth prevalence increased among the 65–74 year-old cohort to approximately 18% and 4% for shallow and deep pockets respectively. There was little difference in pocket depth prevalence between urban and rural adults.

Although clinical loss of attachment is more difficult to measure than pocket depth, attachment loss measures do provide an improved overall estimate of adverse periodontal status [58]. Attachment loss is a historical reflection of periodontal damage throughout a lifetime and does not necessarily indicate that periodontitis is active at the time of assessment. Moreover, delineations between moderate or severe periodontitis have been somewhat fluid. For instance, using data from NHANES III, a variety of researchers have suggested that severe periodontitis is characterized by having at least one tooth with a pocket depth measure of 6 mm or greater whereas others have defined advanced periodontitis as having at least one tooth with 3 mm or greater attachment loss; moderate periodontitis has been defined as having at least one site with pocket depths between 4–5 mm [59-61].

Using data from NHANES III (1988–94) to investigate acute-phase inflammatory markers and periodontitis, Slade has suggested that pocket depth is most likely associated with active periodontal disease [62]. Arbes and coworkers classified individuals with at least 1 periodontal site with concomitant measures of 3 mm of attachment loss and 4 mm of pocket depth as having active periodontal disease [40]. For purposes of this study, we defined "moderate-to-advanced" periodontitis at a lower threshold case definition for disease using Arbes and coworkers' criteria. We also classified individuals with "severe" periodontitis at a higher threshold case definition requiring individuals to have at least one periodontal site with concomitant measures of 4 mm of attachment loss and 5 mm of pocket depth. Although, men were more likely to have more sites of advanced attachment loss compared to women, there was little gender difference in the prevalence of periodontitis at either the lower or upper case definitions for the Linzhou study group.

In this study, NHANES oral health protocols were used in a larger study which was evaluating methods for early detection of esophageal squamous cell cardinoma. ESCC is among the least curable cancers, with 5-year survival rates estimated to be around 10% [63], whereas 5-year survival rates for oral cancer are approximately 50% [64]. The relationship between oral health and risk factors for oral-pharyngeal cancers are well known [65]. Although in many parts of the world the main determinants for both oral cancer and ESCC are tobacco use and alcohol consumption [65,66], the linkage between oral health and ESCC is unclear. In this high risk area of China, the primary risk factors for ESCC are poor nutrition, family history, and other unknown risk factors [67], and oral cancer is uncommon.

More than 20 years ago, a relationship between microorganisms in the oral cavity and those isolated from esophageal carcinomas was suggested [68]. Advances in the understanding of oral microbial ecology have recently supported the notion that dental plaque is an oral biofilm [69] and bacterial biofilms are a common source for chronic infections [70]. Poor oral hygiene contributes to the development of dental plaque, periodontitis, and dental caries [71]. It has been postulated that poor oral hygiene mediates bacterial load and that "overgrowth" of specific bacterial types on teeth may explain many associations seen between poor dental health and systemic diseases [72]. Poor oral hygiene may also contribute to the formation of nitrosamines in the oral cavity [73] and intra-oral nitrate-reducing activity may contribute to the majority of overall nitrosamine exposure in humans [74]. Because nitrosamines are known carcinogens, untreated caries and periodontal disease should be important considerations when exploring cancer risk factors in this study population, which has very high rates of esophageal cancer. After adjusting for a number of potential confounders, the results from our Linzhou study group indicated that poor oral health was associated with esophageal dysplasia (OR = 1.59; 95% CI 1.06, 2.39).

For this report, poor oral health was derived from periodontal status and caries experience. Caries experience (DMFT) is a composite measure comprising of untreated caries (decay), treated caries (fillings), and tooth loss due to disease. Although preliminary findings indicated that DMFT was related to the presence of esophageal dysplasia, subsequent findings showed that this relationship was driven by the missing component (M) of this index. An earlier report from this same region of China suggested that tooth loss was associated with incident esophageal and gastric cancers [24]. In a fuller exploration of risk factors for squamous esophageal dysplasia, a multivariate model also suggested that subjects who had lost more, but not all, of their teeth had a higher prevalence of dysplasia [25]. Tooth loss also has been reported to be associated with oral leukoplakia and oral cancer [26], with pancreatic cancer [75], with gastric cancer in smokers [76], and with esophageal dysplasia [77].

There are some study issues to consider that affect the interpretation of our results. The CSS2 study group does not correspond to a random sample of the general population in the Linzhou area. With a nonsystematic random selection of participants, there may have been a potential bias towards the selection of less healthy study participants. Another potential bias may have been the selection of individuals with certain demographic characteristics. Except for a slightly lower male-to-female ratio, the socio-demographic characteristics of the CSS2 study group were comparable with the general population in this region of China. Nevertheless, the external validity of the CSS2 findings is limited.

Partial-mouth exams, like the NHANES periodontal assessment, may underestimate sites with periodontal disease, particularly the more severe conditions [78-80]. The magnitude of underestimation of disease using any partial-mouth exam is dependent upon the prevalence of the disease in the group under study. When prevalence is low, the degree of underestimation is greater. However, CPI will also underestimate disease severity[81] and the use of CPI to assess for periodontal disease status is controversial [82].

There are additional assumptions and caveats underlying the statistical methods we elected to use. Although the study group was not a systematic random sample, we used parametric statistical tests to test for differences between subgroups in the CSS2 sample. We made this determination following an evaluation for data quality. We also performed non-parametric statistical tests and found that overall estimates and measures of significance were similar to those produced by parametric testing.

There are numerous advantages to using NHANES oral health assessments protocols in a clinical study: there is enhanced comparability of findings across a variety of study designs; there is improved documentation of baseline oral health status for longitudinal studies investigating the development of oral diseases and conditions; and there are enhanced options for aggregating collected data for testing different concepts of risk indicator exposure/outcomes. For similar reasons, the Women's Interagency HIV Study (WIHS), a multi-center clinical study in the U.S., uses NHANES oral health protocols [83]. The NHANES methodology may not be suitable for all dental research activities, such as some clinical trials, but these methods should be suitable for exploring many systemic interrelationships.

## Conclusion

Findings from this study represent the first attempt to collect oral health data employing NHANES protocols outside of the U.S. These findings suggest that tooth loss, dental caries, and periodontal disease are important oral health issues in Linzhou, China. Moreover, these findings suggest that poor oral health may be associated with esophageal dysplasia. In this study, we used a definition of poor oral health that is a composite of periodontal attachment loss and dental caries experience, which are measures of oral diseases that are promulgated by specific oral bacteria. Our findings do show that there is no association between esophageal dysplasia and edentulism.

## Authors' contributions

BAD participated in the study design; coordinated data collection, analysis, and interpretation of data; and contributed to the drafting and review of the manuscript. RW provided oversight of data collection, contributed to the manuscript conception, and contributed to drafting the article and final review of the manuscript. RL participated in collection and interpretation of the data, and contributed to the drafting of the manuscript. WQW participated in the study design, provided oversight of data collection and significantly contributed to the review of the manuscript. SMD and YLQ conceived of the study and significantly contributed to the review of the manuscript. GQW, CCA, and MJR participated in the study design and significantly contributed to the review of the manuscript. XL and WC participated in the data collection, the drafting of the article and the final review of the manuscript. All authors (except for RL) read and approved the final manuscript. RL was unable to review and provide comments on the final revisions of this manuscript due to her unexpected death on 28 April 2005 as a result of cancer.

## Pre-publication history

The pre-publication history for this paper can be accessed here:


